# A case series of group-based ketamine-assisted psychotherapy for patients in residential treatment for eating disorders with comorbid depression and anxiety disorders

**DOI:** 10.1186/s40337-022-00588-9

**Published:** 2022-05-06

**Authors:** Reid Robison, Adele Lafrance, Madeline Brendle, Michelle Smith, Claire Moore, Sachin Ahuja, Scott Richards, Nicole Hawkins, Erin Strahan

**Affiliations:** 1 Center for Change, Orem, UT USA; 2 Novamind Inc., Draper, UT USA; 3 Department of Psychiatry, University of Utah School of Medicine, Salt Lake City, UT USA; 4 Emotion Science, Denver, CO USA; 5 Department of Pharmacotherapy, University of Utah College of Pharmacy, Salt Lake City, UT USA; 6 Wilfrid Laurier University, Brantford, ON Canada

**Keywords:** Ketamine, Psychotherapy, Feeding and eating disorders, Depression, Anxiety, Case series

## Abstract

**Background:**

Depression and anxiety outcome measures, safety/tolerability, patient satisfaction, and ease of implementation of group-based ketamine-assisted psychotherapy (G-KAP) delivered to patients in intensive residential eating disorder (ED) treatment were assessed.

**Case presentation:**

This study reports on five participants with a diagnosis of an ED and comorbid mood and anxiety disorders who received weekly intramuscular ketamine injections in a group setting over 4 weeks. Measures of anxiety (GAD-7) and depression (PHQ-9) were administered pre-dose, 4-h post-dose, and 24-h post dose. Four of the 5 participants experienced clinically significant improvements on the PHQ-9 score (i.e., change greater than 5) while 2 of the 5 participants experienced clinically significant improvements on the GAD-7 score (i.e., change greater than 4) from pre-dose to 24-h post-dose after the last ketamine session. Dosing sessions were well tolerated, and no serious adverse events were reported. Clinical observations and participant reports corroborated improvements in depression and anxiety symptoms, good tolerability of ketamine treatment, and practical implementation of the G-KAP protocol in a residential ED treatment center.

**Conclusions:**

This study suggests the potential utility of G-KAP as an adjunct to intensive, specialized ED treatment. Overall, this novel, cross-diagnostic intervention warrants future research to further explore its appropriateness in a treatment setting.

## Introduction

Ketamine is a dissociative arycyclohexylamine derivative and an antagonist of the N-methyl-D-aspartate (NMDA)-receptor [1, 2]. The hypothesized mechanism of action for ketamine’s antidepressant effects include direct effects of NMDA receptor antagonism as well as additional mechanisms which may include ketamine metabolites [1]. Ketamine was FDA-approved in the 1970s as an anesthetic agent and has been found to be safe and effective in the treatment of depression in the context of a number of controlled studies [3–6]. In a recent systematic review study of 35 randomized controlled trials of ketamine for unipolar depression, intravenous ketamine was effective in 70% of the included studies (21/30) and oral and intranasal ketamine were demonstrated to be effective in five studies total [6]. Across various studies, the most common physiological adverse effects reported included dissociation and slight increases in blood pressure which have been observed to resolve upon elimination of ketamine from the system [1, 7, 8]. The dissociative effects of ketamine appear to be dose-dependent and are commonly described as having an extracorporeal sensation that can be accompanied by synesthetic hallucinations, even when given at a subanesthetic doses [1, 9]. While ketamine’s psychopharmacological effects on depression are rapid-acting and transient, there is a growing interest in the possibility of prolonging the antidepressant effects by combining ketamine with psychotherapy (i.e., ketamine-assisted psychotherapy, KAP) [4, 10, 11]. Ketamine’s NMDA receptor antagonism also induces neuroplasticity, facilitating the formation of new neural connections and restructuring of thought patterns, making ketamine a potentially powerful substance as an adjunct to psychotherapy [12, 13].

In addition to major depressive disorder, studies point to the salutary effects of ketamine and KAP in the context of other mental health disorders including bipolar disorder, substance dependence, and anxiety disorders [5, 14–18]. For example, Glue et al. [15] conducted a double-blind, psychoactive-controlled ascending dose study in 12 patients with treatment-resistant generalized anxiety and social anxiety disorders. Not only was ketamine safe and well-tolerated among participants, improvements in anxiety ratings occurred within an hour of ketamine dosing and persisted for up to 1 week.

Preliminary evidence also exists with respect to ketamine’s potential for those struggling with eating disorders (ED) [19–24]. Specifically, Mills et al. [19] administered infusions of 20 mg per hour of ketamine for 10 h to treat 15 patients with chronic and severe ED.^1^ Sixty percent of those treated showed prolonged remission on a measure of compulsion when treated with several ketamine infusions over 3 weeks, whereas there was no significant response to at least 5 ketamine treatments for the remainder of the sample. One of the hypotheses for the non-response related to the compulsive drive being re-established too soon after the infusion. As such, with combined psychotherapy during the period of increased neuroplasticity, stronger results could be expected. More recently, a case series by Schwartz et al. [21] reported on four patients with enduring ED and treatment-resistant depression treated with repeat intramuscular (IM) ketamine at 4–6 week intervals over 12 + months. The ketamine treatment reduced depression in all four cases; however, the effects on ED symptoms were modest. Additionally, two recent case reports reported promising evidence of ketamine therapy for anorexia nervosa and bulimia nervosa [20, 23]. The case report by Scolnick et al. [20] described an instance where an individual with severe and enduring anorexia nervosa received a short series of titrated intravenous ketamine infusions, combined with dietary interventions, leading to complete remission for a period of 6 months. Additionally, the case report by Ragnhildstveit et al. [23] reported on an individual with extreme and enduring bulimia nervosa treated with 3 courses of KAP over 3 months, with each course consisting of 6 sessions twice weekly, also leading to complete and sustained remission for over 1 year to date [23].

While the results among sufferers of EDs can be regarded as modest, they remain promising and point to the need for further research, in particular to treat psychiatric comorbidities. Specifically, the majority of those struggling with an ED also struggle with symptoms of depression and anxiety, and these symptoms often predate the onset of the ED [25–27]. In a recent study conducted by Martín et al. [28] among 520 patients with an ED, it was found that depressive and anxiety symptoms were related to ED symptoms, health status, and medical comorbidity, among other variables [28]. In fact, Fewell et al. [29] recommend a focus on depressive and worry symptoms among ED patients in higher levels of care on the basis of their research which showed that these features predicted higher levels of ED symptomatology and psychological impairment at discharge and at 1-year follow-up. This is important when considering the lack of reliable responses to treatment and high relapse rates among those who suffer from EDs [22, 30, 31].

Considering this body of research and the need for more effective ED treatments across the continuum of illness severity, it is critical to explore safe and promising new treatments targeting comorbid symptoms of depression and anxiety. Given the uniqueness of ketamine as a potential antidepressant, anxiolytic, and a catalyst for psychotherapy, we conducted a prospective case series study to explore depression and anxiety outcomes, safety, and patient satisfaction of group-based KAP (G-KAP) among participants in residential intensive treatment for an ED.

## Case presentation

### Participants

The sample consisted of five female patients, age ranging from 22 to 43, who were receiving treatment at a residential ED treatment center. The patients were referred to participate in G-KAP by their primary therapist, and subjects were screened by the residential treatment center’s Medical Director for the medical and psychiatric evaluation. Using DSM-5 criteria, three participants were diagnosed with Anorexia Nervosa (restricting subtype), one participant was diagnosed with Anorexia Nervosa (binge-purge subtype), and one participant was diagnosed with Bulimia Nervosa. All participants had a comorbid diagnosis of major depressive disorder, recurrent (from moderate to severe) and met criteria for either generalized anxiety disorder (n = 3) or post-traumatic stress disorder (n = 2). Current medications are listed in Table 1. Additionally, all participants had prior ED treatment, including treatment in the following settings for all participants: Inpatient, Residential Treatment Center, Partial Hospitalization Program, Intensive Outpatient Program. All participants had received several previous psychiatric medication trials (Table 1).

**Table 1:** Demographic and clinical characteristics of the 5 G-KAP participants

Participant	Gender	Age	Primary diagnosis	Secondary diagnoses	Current medications	Past medication trials
1	F	22	AN, restricting type	MDD, moderate, recurrent GAD	Venlafaxine ER 300 mg PO QAM for mood Temezapem 15–30 mg PO QHS PRN for insomnia	Fluoxetine Sertraline Bupropion Fluvoxamine Risperidone
2	F	28	BN	MDD, severe, recurrent PTSD	Fluvoxamine 100 mg QAM and 200 mg QHS for mood/anxiety Gabapentin 300 mg PO three times a day (TID) for anxiety Aripiprazole 5 mg PO QAM for mood Trazodone 100–150 mg PO QHS PRN for insomnia	Aripiprazole Fluvoxamine Gabapentin Trazodone Fluoxetine Prazosin
3	F	43	AN, restricting type	MDD, severe, recurrent GAD	Alprazolam 0.25–0.5 mg PO QD PRN for anxiety Buspirone 30 mg PO QAM for anxiety Desvenlafaxine 50 mg PO QAM for depression Quetiapine 12.5–25 mg PO QHS PRN for insomnia	Desvenlafaxine Alprazolam Fluoxetine Quetiapine Buspirone
4	F	37	AN, binge-purge type	MDD, severe, recurrent GAD	Aripiprazole 10 mg PO QHS for mood, clonazepam 0.5 mg PO QD PRN for anxiety Duloxetine 90 mg PO QAM for depression Lamotrigine 100 mg PO BID for mood Modafinil 100 mg PO QAM for depression Prazosin 2 mg PO QHS for PTSD Temazepam 15–30 mg PO QHS PRN for insomnia	Aripiprazole Clonazepam Duloxetine Lamotrigine Temazepam Modafinil
5	F	31	AN, restricting type	MDD, severe, recurrent PTSD	Aripiprazole 15 mg PO QHS for mood clonazepam 0.5–1 mg PO QD PRN for anxiety Duloxetine 120 mg PO QHS for depression Prazosin 5 mg PO QHS for PTSD Propranolol 5–10 mg PO BID PRN for anxiety Temazepam 15–30 mg PO QHS PRN for insomnia	Aripiprazol Duloxetine Clonazepam Prazosin Propanolol, temazepam Zolpidem Lamotrigine Quetiapine

*AN* Anorexia Nervosa; *BN* Bulimia Nervosa; *MDD* Major Depressive Disorder; *GAD* Generalized Anxiety Disorder; *PTSD* Post-Traumatic Stress Disorder; *PO* by mouth; *QAM* once a day in the morning; *QHS* every night at bedtime; *PRN* as needed; *TID* three times a day; *QD* once a day; *BI* twice a day

### Setting

The study occurred in a well-respected residential ED program that serves up to 30 patients. The purpose of the residential ED treatment center is to provide professional, compassionate care in a safe environment. Group therapy includes debriefing and goal setting, skills group (dialectical behavioral therapy/cognitive behavioral therapy), balance and awareness group, open process group, group movement therapy (i.e., yoga), art recreational therapy group, body image group, self-esteem group, community meeting, spirituality/12-step group, and relapse prevention group. Individual therapy for patients includes psychotherapy sessions with a licensed therapist two times per week including one family therapy session, two sessions per week with a registered dietician, and medical and psychiatric rounding. Meals and snacks are all supervised, and a 24-h nurse is available for patient care.

### Procedure

The G-KAP treatment took place in groups of four, once a week on Monday mornings over a 4-week period. Staff present were the medical director (principal investigator) and two staff nurses. The group treatment room included reclining sofas and eyeshades, headphones, and music were provided to each participant. Before each IM ketamine dose (pre-dose) and four hours after each dose, and 24-h after each dose, participants completed self-reported measures of depression and anxiety—the Patient Health Questionnaire-9 (PHQ-9) and the Generalized Anxiety Disorder Scale-7 (GAD-7), respectively. These measures were administered as a part of routine clinical practice to monitor treatment effectiveness.

For the first session, each participant received an initial dose of 25 mg IM ketamine in the group treatment room. After the first G-KAP session, the IM ketamine dose varied according to participant needs and was increased each session as tolerated (Table 2). The typical dosing schedule is 25 mg, 25–40 mg, 40–60 mg, and 40–100 mg for dosing sessions 1, 2, 3, and 4, respectively. Individual dosing is based on response and exploration of the patient’s comfort level and experience to the first dose of ketamine treatment. Thus, the clinician increased the dose as tolerated based on the first dose of ketamine treatment for each participant individually. After IM ketamine administration, participants were carefully monitored every 15 min. Two nurses and physician remained present throughout. Individual check-in and support were provided as needed throughout the dosing session. Brief processing took place a group after approximately 90 min post-dosing. Patients then returned to the ED residential unit and resumed participation in unit programming.

**Table 2:** Intramuscular ketamine dosage for sessions 1–4

Participant	Dose 1 (mg)	Dose 2 (mg)	Dose 3 (mg)	Dose 4 (mg)
1	25	40	50	100
2	25	40	40	40
3	25	25	25	40
4	25	40	60	100
5	25	40	60	80

### Depression and anxiety outcome measures

#### Patient Health Questionnaire-9 (PHQ-9)

The PHQ-9 is a 9-item questionnaire designed to measure the range and severity of symptoms of depression [32]. Participants rate each item on a 4-point Likert scale ranging from “0—Not at all” to “3—Nearly every day.” Participants were asked to indicate the extent to which they had been bothered by these symptoms over the past 2 weeks. Sample items include: “feeling down, depressed or hopeless” and “feeling bad about yourself—or that you are a failure or have let yourself or your family down.” The total PHQ-9 score is calculated as a sum of responses to all 9 questions, with higher scores indicating greater symptoms of depressed mood. The minimal clinically significant change on the PHQ-9 was defined as a 5-point change [33].

#### Generalized Anxiety Disorder Scale-7 (GAD-7)

The GAD-7 is a 7-item questionnaire designed to measure symptoms of anxiety [34]. Participants rate each item on a 4-point Likert scale ranging from “0—Not at all” to “3—Nearly every day.” Participants were asked to indicate the extent to which they had been bothered by these symptoms over the past 2 weeks. Sample items include: “feeling nervous, anxious or on edge” and “feeling afraid as if something awful might happen.” The total GAD-7 score is calculated as a sum of responses to all 7 questions, with higher scores indicating greater symptoms of anxiety. The minimal clinically significant change on the GAD-7 was defined as a 4-point change [35].

### Data analysis

Descriptive statistics were used to present and estimate individual change in pre-dose and post-dose PHQ-9 and GAD-7 scores across the study period for the five participants. Descriptive statistics were also used to summarize vital sign information.

## Results

### Depression and anxiety outcomes—PHQ and GAD-7

**Fig. 1:**
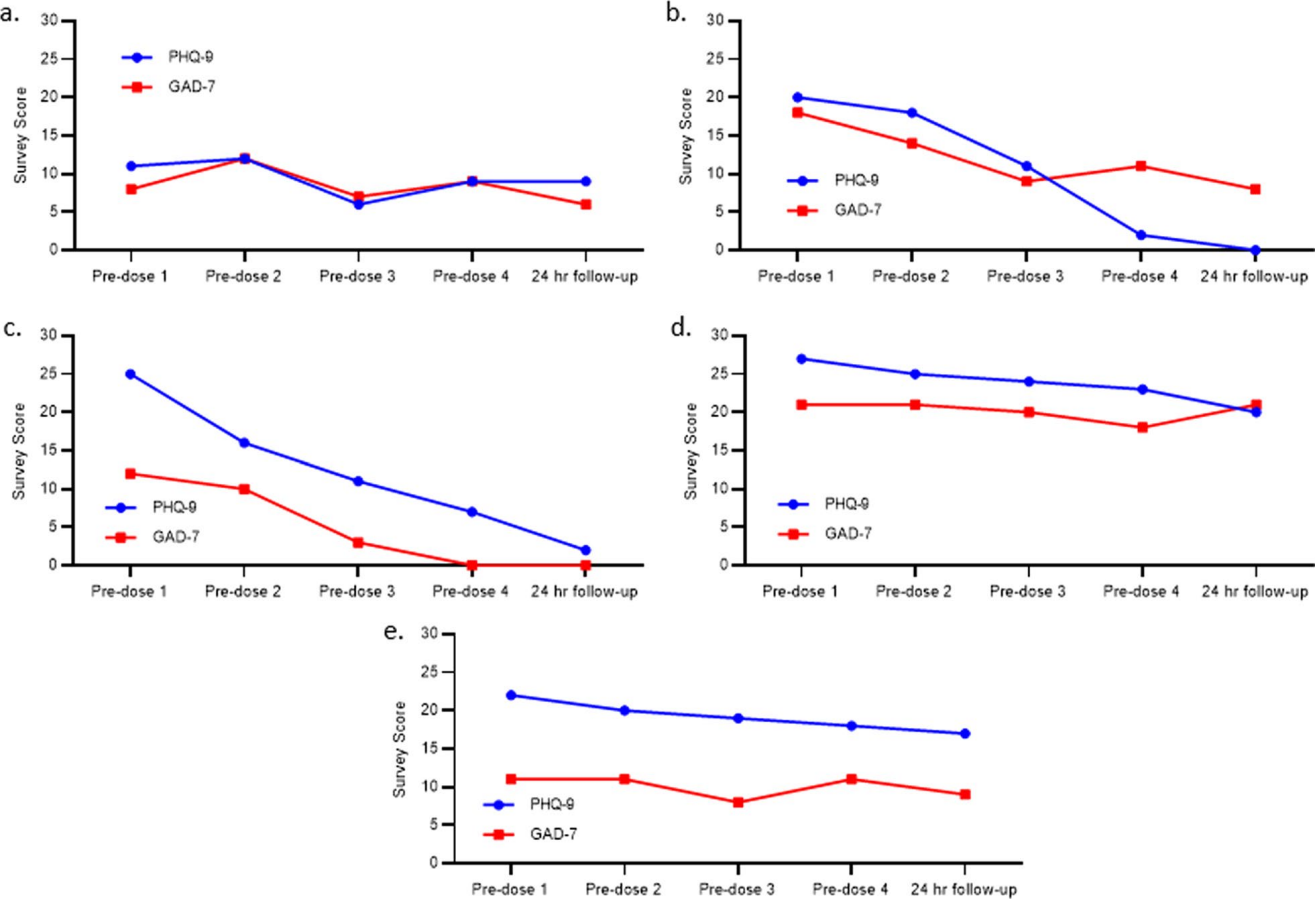
Individual participant PHQ-9 and GAD-7 pre-dose measures for IM ketamine dosing sessions 1–4 over the 4-week study period and 24-h follow-up after the fourth ketmaine dose. (**a**: participant 1, **b**: participant 2, **c**: participant 3, **d**: participant 4, **e**: participant 5)

Each of the participants’ PHQ-9 and GAD-7 scores for pre-dose timepoints across the four treatment sessions and the 24-h follow-up after the last IM ketamine dose are presented individually (Fig. 1a–e). We observed variability in terms of the extent of participant improvement on symptoms of depression and anxiety with IM ketamine treatment over the 4-week study period for the 5 participants. In terms of reduction in the PHQ-9 and GAD-7 scores from pre-dose to the 24-h follow-up after the fourth dose, 4 participants (participant 2, 3, 4, 5) experienced clinically significant improvements on the PHQ-9 while 2 participants (participant 2, 3) experienced a clinically significant improvement on the GAD-7. We observed the largest change in PHQ-9 scores for participant 2 (pre-dose: 20, follow-up: 0) and participant 3 (pre-dose: 25, follow-up: 2). Both participants went from the severe depression category to minimal depression category from pre-dose to the 24-h follow-up. These 2 participants also had the largest change in GAD-7 scores of the 5 participants (participant 2 pre-dose: 18, follow-up: 8; participant 3 pre-dose: 12, follow-up 0). Participant 2 went from the severe anxiety category to mild anxiety while participant 3 went from the moderate anxiety category to minimal anxiety. Participants 1, 4, and 5 had modest changes in their PHQ-9 and GAD-7 scores from pre-dose to the 24-h follow-up. For the PHQ-9, participant 1’s score reduced from 11 to 9, participant 4’s score reduced from 27 to 20, and participant 5’s score reduced from 22 to 17. For the GAD-7, participant 1’s score reduced from 8 to 6, participant 4’s score stayed the same at 21, and participant 5’s score reduced from 11 to 9.

### Blood pressure and heart rate

During the four ketamine dosing sessions for the five participants, blood pressure slightly decreased while heart rate increased, on average, from pre-dose to 30-min post-dose and from 30-min post-dose to one-hour post-dose. Mean blood pressure for the participants was measured as 102/67 (SD 9/10) mmHg pre-dose, 102/64 (SD 10/12) mmHg 30-min post-dose, and 94/55 (SD 7/5) 1-h post dose across the four dosing sessions. Mean heart rate fell by approximately 3 beats per minute (BPM) from pre-dose (107, SD 15) to 30-min post-dose (82, SD 11), and fell by approximately 7 BPM from 30-min post-dose to one-hour post dose (75, SD 9.3).

### Safety and tolerability

The attending psychiatrist monitored treatment response of all participants. According to this report, the acute effects of ketamine were well-tolerated by all patients, and no serious or unexpected adverse events occurred. Patients were offered a dose of 4–8 mg of Zofran to prevent nausea before administering ketamine based on nausea history and past response to this medicine. The most common non-dissociative adverse events were nausea, dizziness, and blurred vision. No patients reported symptoms of cystitis or memory problems at any time during maintenance treatment.

### Patient satisfaction

We inquired regarding patient satisfaction in order further inform ketamine programs for EDs at a residential ED treatment center. Participants were sent an online survey to gather their satisfaction and experience with G-KAP at the residential ED treatment center. Of the 5 participants, survey responses were returned by 3 participants. Satisfaction was rated by both yes/no questions and 5-point Likert scales. Questions targeted participant perceptions of safety, efficacy, and satisfaction. Sample questions included: “Did you feel benefit from participation in the Ketamine”, “Do you feel you were harmed by participation in these treatments?” and “Do you think additional ketamine sessions would have been helpful?”.

All participants who completed the satisfaction survey reported benefit from participation in the ketamine treatments, with a mean rating of the degree of benefit 3.67/5 (SD 0.94). None of the participants surveyed reported being harmed by their participation. Two of the three participants who completed the survey reported improvement in ED thoughts or behaviors as a result of the ketamine treatments. The participant that reported their ED symptoms did not improve with ketamine treatment stated, “Not sure if it helped with ED. I think it did help with my baseline depression, but hard to tell if that was the treatment or if it was being in 24 h care.” One participant that reported their ED symptoms improved with ketamine treatment stated, “My ED symptoms were greatly improved during the short treatment and for a couple hours afterward. After that, they basically came back, but it was helpful to catch the vision of what life could be like without them.” Another participant added, “The effect of easing my rigidity toward ED rules was extremely helpful at making me face challenges that were normally too anxiety provoking to face alone.”

All three participants stated that the ketamine dose was both safe and appropriate. One participant commented: “I do think I could have received more benefit from a higher dose, especially with my history of having a pretty high tolerance for (therapeutic) substances.” Of note, all three of the participants felt that additional ketamine treatments after the study completion would have been helpful (4–6 additional sessions).

Two participants completed a summary of their experience with ketamine in an ED treatment center setting in respect to their ED or in general. One participant wrote, “Trying Ketamine at the residential ED treatment center allowed me to see the possibility of a life I could have. While the effects did not last, my very first experience snapped me out of a state of life-long, deep disconnection that I didn't even know I had been experiencing. Suddenly, I was able to live in the world in the way people had always described it. Though I am still trying to figure out how to attain that level of connection after catching a glimpse, that one experience was so essential. I could finally feel hunger and fullness cues. I felt what it's like to live in a body, instead of living a short distance from it. I felt connected to others and genuinely cared about their well-being. I felt human for the first time in a long time.” Another participant stated, “After the 4 week program, my depression was better than it's been for 18 years.”

### Medical staff feedback

The G-KAP protocol was determined implementable in a residential ED treatment center setting by the medical staff. The group setting and shared experience was a positive aspect of the treatment as reported by the participants. The clinicians and nurses at the residential ED treatment center found the treatment was integrated successfully to their daily programming and did not interfere or negatively affect the subjects’ participation in the care and peer interactions at the treatment center. The experience was rewarding for both patients and staff, and there was continued enthusiasm and support for the program by management of the ED treatment center.

## Discussion and conclusions

This case series is the first of its kind to explore depression and anxiety outcome measures, safety, and patient satisfaction of a G-KAP protocol to treat mood and anxiety symptoms of patients in intensive ED treatment. Overall, there were no major safety concerns, participant feedback was positive, and most participants improved in anxiety and/or depression symptoms. With respect to depression and anxiety outcome measures, 4 participants showed clinically significant improvement in depression symptoms (participant 2, 3, 4, 5) while 2 participants showed clinically significant improvement in anxiety symptoms (participant 2, 3) from pre-dose to 24-h follow-up after the fourth ketamine dosing session. However, not all participants improved in depression/anxiety symptoms. For the patients who did not experience a minimal clinically significant improvement in their PHQ-9 and GAD-7 scores, additional ketamine treatment sessions or an increase in dose may have been needed, as was also noted in participant feedback. A conventional course of ketamine for sustained benefit typically requires multiple infusions at a high-frequency during an induction period, typically 6 sessions over 2–3 weeks, followed by lower-frequency treatment during maintenance treatment [11, 36].

The seemingly rapid response in mood to ketamine treatments observed in some cases is congruent with previous studies of ketamine for depression. For example, in a meta-analysis by Kishimoto et al. [4], the antidepressant benefits were apparent at 40 min, peaked at day 1, and were lost at days 10–12. The improvements may have been sustained for the participants in our study given the treatment setting in which the ketamine protocols took place. Specifically, in addition to acute anti-depressive and anxiolytic effects, ketamine is thought to induce a state of increased neuroplasticity, and therefore participation in the residential treatment programming could have influenced the extent to which the ketamine treatments were effective and vice versa. Therefore, it is possible that a ketamine regime could be used to enhance treatment response more globally, and in particular for those who struggle to benefit from conventional ED treatment.

In terms of safety, the acute effects of ketamine were well-tolerated by all participants, and no serious or unexpected adverse events occurred. In addition to good tolerability, the protocol was implementable in that ketamine was integrated successfully into treatment-as-usual in a residential care setting, and participants generally reported the experience as being a positive and meaningful part of their course of treatment. Informal feedback from staff at the treatment center supported this evaluation. In fact, our study suggests that not only is it possible to offer ketamine treatments in group settings, the participants seemed to appreciate the shared experience, much like the therapeutic factors observed in conventional group therapy settings [37].

We are hopeful that the results of this study can contribute to the growing body of literature relating to new adjunctive treatments in ED care, including ketamine treatments. To our knowledge, this is the first group-based ketamine intervention in a higher level of care for ED. Overall, this cross-diagnostic intervention seems a worthwhile object of future study to assess improvement in depression and anxiety symptoms of ED patients in residential treatment programs. One participant also attributed improvements in ED symptoms to her involvement in this adjunctive treatment. Whether these reductions prove short- or long-term relief to patients and/or create neuro-cognitive conditions for greater response to ED treatment, the fact that this intervention was short-term, required few additional resources, and was delivered to patients with varied symptom profiles is reason for optimism.

Despite its important contribution to the field, the contents of this report must be interpreted in the context of its limitations. Although this G-KAP intervention appears to have benefitted these participants, firm conclusions about its effectiveness cannot be drawn due to the nature of the study. Specifically, the study was not adequately powered or structured to determine duration of antidepressant and anxiolytic benefits. We cannot attribute the improvements in depression and anxiety symptoms to ketamine because the participants were also receiving intensive treatment in a residential center and there was no placebo group. This is an important area of future research in this population, as the psychopharmacological effects of ketamine on mood and anxiety are generally considered to be temporary, but little is known about the potential of ketamine in combination with intense treatment to prolong these effects. The generalizability of our results is also limited by our small sample size, and patient satisfaction follow-up data was provided by 3 of the 5 participants.

For future research, it is recommended that additional ketamine interventions continue to be explored as an adjunct to intensive treatment for EDs. There were no major concerns with implementing this G-KAP treatment in a residential ED treatment center that would prohibit the design of a larger feasibility or pilot study to be conducted in the future. In addition to a larger feasibility or pilot study, future studies should explore the differential impact of the various treatment doses as well as the relationships among different variables (such as diagnosis, illness duration, and length of time in treatment) and additional outcomes, including eating-disorder related symptoms. Finally, follow-up studies will be essential in order to better understand the long-term outcomes related to this intervention. Although limited conclusions can be drawn about treatment efficacy from this study, G-KAP represents a promising paradigm for depression and anxiety symptoms in individuals with ED that warrants further research.

## Data Availability

The datasets used and/or analyzed during the current study are available from the corresponding author on reasonable request.

## Abbreviations

DSM-5: Diagnostic and Statistical Manual of Mental Disorders (DSM-5)
ED: Eating disorder
G-KAP: Group ketamine-assisted psychotherapy
KAP: Ketamine-assisted psychotherapy
NMDA: *N*-Methyl-d-aspartate

## Footnotes

1 Twenty milligrams twice daily of nalmefene was administered to prevent loss of consciousness during the ketamine treatment.

## References

[1] Zanos P, Gould TD (2018). Mechanisms of ketamine action as an antidepressant. Mol Psychiatry.

[2] Ho JH, Dargan PI, Brent J, Burkhart K, Dargan P, Hatten B, Megarbane B, Palmer R (2016). Arylcyclohexamines (ketamine, phencyclidine, and analogues). Critical care toxicology.

[3] Fond G, Loundou A, Rabu C, Macgregor A, Lançon C, Brittner M (2014). Ketamine administration in depressive disorders: a systematic review and meta-analysis. Psychopharmacology.

[4] Kishimoto T, Chawla JM, Hagi K, Zarate CA, Kane JM, Bauer M (2016). Single-dose infusion ketamine and non-ketamine N-methyl-d-aspartate receptor antagonists for unipolar and bipolar depression: a meta-analysis of efficacy, safety and time trajectories. Psychol Med.

[5] Kraus C, Rabl U, Vanicek T, Carlberg L, Popovic A, Spies M (2017). Administration of ketamine for unipolar and bipolar depression. Int J Psychiatry Clin Pract.

[6] Memon RI, Naveed S, Faquih AE, Fida A, Abbas N, Chaudhary AMD (2020). Effectiveness and safety of ketamine for unipolar depression: a systematic review. Psychiatr Q.

[7] Clements JA, Nimmo WS, Grant IS (1982). Bioavailability, pharmacokinetics, and analgesic activity of ketamine in humans. J Pharm Sci.

[8] Szarmach J, Cubała WJ, Włodarczyk A, Wiglusz MS (2019). Short-term ketamine administration in treatment-resistant depression: focus on cardiovascular safety. Psychiatr Danub.

[9] Kurdi M, Theerth K, Deva R (2014). Ketamine: current applications in anesthesia, pain, and critical care. Anesth Essays Res.

[10] Hasler G (2020). Toward specific ways to combine ketamine and psychotherapy in treating depression. CNS Spectr.

[11] Dore J, Turnipseed B, Dwyer S, Turnipseed A, Andries J, Ascani G (2019). Ketamine assisted psychotherapy (KAP): patient demographics, clinical data and outcomes in three large practices administering ketamine with psychotherapy. J Psychoactive Drugs.

[12] Ingram R, Kang H, Lightman S, Jane DE, Bortolotto ZA, Collingridge GL (2018). Some distorted thoughts about ketamine as a psychedelic and a novel hypothesis based on NMDA receptor-mediated synaptic plasticity. Neuropharmacology.

[13] Yao N, Skiteva O, Zhang X, Svenningsson P, Chergui K (2018). Ketamine and its metabolite (2R,6R)-hydroxynorketamine induce lasting alterations in glutamatergic synaptic plasticity in the mesolimbic circuit. Mol Psychiatry.

[14] Abdallah CG, Roache JD, Averill LA, Young-McCaughan S, Martini B, Gueorguieva R (2019). Repeated ketamine infusions for antidepressant-resistant PTSD: methods of a multicenter, randomized, placebo-controlled clinical trial. Contemp Clin Trials.

[15] Glue P, Medlicott NJ, Neehoff S, Surman P, Lam F, Hung N (2020). Safety and efficacy of extended release ketamine tablets in patients with treatment-resistant depression and anxiety: open label pilot study. Ther Adv Psychopharmacol.

[16] Krupitsky EM, Burakov AM, Dunaevsky IV, Romanova TN, Slavina TY, Grinenko AY (2007). Single versus repeated sessions of ketamine-assisted psychotherapy for people with heroin dependence. J Psychoactive Drugs.

[17] Liriano F, Hatten C, Schwartz TL (2019). Ketamine as treatment for post-traumatic stress disorder: a review. Drugs Context.

[18] Rodriguez CI, Kegeles LS, Levinson A, Feng T, Marcus SM, Vermes D (2013). Randomized controlled crossover trial of ketamine in obsessive-compulsive disorder: proof-of-concept. Neuropsychopharmacology.

[19] Mills IH, Park GR, Manara AR, Merriman RJ (1998). Treatment of compulsive behaviour in eating disorders with intermittent ketamine infusions. QJM.

[20] Scolnick B, Zupec-Kania B, Calabrese L, Aoki C, Hildebrandt T (2020). Remission from chronic anorexia nervosa with ketogenic diet and ketamine: case report. Front Psychiatry.

[21] Schwartz T, Trunko ME, Feifel D, Lopez E, Peterson D, Frank GKW (2021). A longitudinal case series of IM ketamine for patients with severe and enduring eating disorders and comorbid treatment-resistant depression. Clin Case Rep.

[22] Keeler JL, Treasure J, Juruena MF, Kan C, Himmerich H (2021). Ketamine as a treatment for anorexia nervosa: a narrative review. Nutrients.

[23] Ragnhildstveit A, Jackson LK, Cunningham S, Good L, Tanner Q, Roughan M (2021). Case report: unexpected remission from extreme and enduring bulimia nervosa with repeated ketamine assisted psychotherapy. Front Psychiatry.

[24] Martinotti G, Chiappini S, Pettorruso M, Mosca A, Miuli A, Di Carlo F (2021). Therapeutic potentials of ketamine and esketamine in obsessive-compulsive disorder (OCD), substance use disorders (SUD) and eating disorders (ED): a review of the current literature. Brain Sci.

[25] Blinder BJ, Cumella EJ, Sanathara VA (2006). Psychiatric comorbidities of female inpatients with eating disorders. Psychosom Med.

[26] Bulik CM (2002). Anxiety, depression and eating disorders.

[27] Simonetta M, Laura Dalla R, De Giulia I, Tania M, Maria V, Edoardo G (2018). Anorexia nervosa and comorbid psychopathology. Endocr Metab Immune Disord - Drug Targets.

[28] Martín J, Arostegui I, Loroño A, Padierna A, Najera-Zuloaga J, Quintana JM (2019). Anxiety and depressive symptoms are related to core symptoms, general health outcome, and medical comorbidities in eating disorders. Eur Eat Disord Rev.

[29] Fewell LK, Levinson CA, Stark L (2017). Depression, worry, and psychosocial functioning predict eating disorder treatment outcomes in a residential and partial hospitalization setting. Eat Weight Disord – Stud Anorex Bulim Obes.

[30] Khalsa SS, Portnoff LC, McCurdy-McKinnon D, Feusner JD (2017). What happens after treatment? A systematic review of relapse, remission, and recovery in anorexia nervosa. J Eat Disord.

[31] Treasure J, Claudino AM, Zucker N (2010). Eating disorders. The Lancet.

[32] Kroenke K, Spitzer RL (2002). The PHQ-9: a new depression diagnostic and severity measure. Psychiatr Ann.

[33] Kroenke K (2012). Enhancing the clinical utility of depression screening. CMAJ: Can Med Assoc J = journal de l'Association medicale canadienne.

[34] Spitzer RL, Kroenke K, Williams JBW, Löwe B (2006). A brief measure for assessing generalized anxiety disorder: the GAD-7. Arch Intern Med.

[35] Toussaint A, Hüsing P, Gumz A, Wingenfeld K, Härter M, Schramm E (2020). Sensitivity to change and minimal clinically important difference of the 7-item Generalized Anxiety Disorder Questionnaire (GAD-7). J Affect Disord.

[36] Sanacora G, Frye MA, McDonald W, Mathew SJ, Turner MS, Schatzberg AF (2017). A consensus statement on the use of ketamine in the treatment of mood disorders. JAMA Psychiat.

[37] Yi D (1995). The theory and practice of group psychotherapy.

